# A new *Haptoclinus* blenny (Teleostei, Labrisomidae) from deep reefs off Curaçao, southern Caribbean, with comments on relationships of the genus

**DOI:** 10.3897/zookeys.306.5198

**Published:** 2013-06-04

**Authors:** Carole C. Baldwin, D. Ross Robertson

**Affiliations:** 1 Department of Vertebrate Zoology, National Museum of Natural History, Smithsonian Institution, Washington, DC 20560; 2 Smithsonian Tropical Research Institute, Balboa, Republic of Panamá

**Keywords:** Blenniiformes, submersible, Substation Curaçao, *Haptoclinus apectolophus*, Deep Reef Observation Project (DROP)

## Abstract

A second species of the blenniiform genus *Haptoclinus* is described from deep reefs off Curaçao, southern Caribbean. *Haptoclinus dropi* **sp. n.** differs from the northwestern Caribbean *Haptoclinus apectolophus* Böhlke and Robins, 1974, in having 29 total dorsal-fin elements—III-I-XIII, 12 (vs. 31—III-I-XIV, 13 or III-I-XIII, 14); 19 anal-fin soft rays (vs. 20-21); 12 pectoral-fin rays (vs. 13); 12 precaudal vertebrae (vs. 13); and the first dorsal-fin spine longer than the second (vs. the second longer than the first). It further differs from *Haptoclinus apectolophus* in lacking scales (vs. three-quarters of body densely scaled), in having a distinctive pattern of spotting on the trunk and fins in preservative (vs. no spotting), and in lacking a fleshy flap on the anterior rim of the posterior nostril (vs. flap present). Color in life is unknown for *Haptoclinus apectolophus*, and the color description presented for the new species constitutes the first color information for the genus. Familial placement of *Haptoclinus* remains questionable, but the limited relevant information obtained from morphological examination of the new species provides additional support for a close relationship with the Chaenopsidae. *Haptoclinus dropi* represents one of numerous new teleost species emerging from sampling to 300 m off Curaçao as part of the Smithsonian Institution’s Deep Reef Observation Project (DROP).

## Introduction

Diving to 300 m off Curaçao in the southern Caribbean using Substation Curaçao’s (http://www.substation-Curacao.com) manned submersible *Curasub* is expanding our knowledge of the deep-reef Caribbean fish fauna. Targeted fish specimens are collected with the sub’s two flexible, hydraulic arms, one of which is equipped with a quinaldine-ejection system and the other with a suction hose. Occasionally, small, inconspicuous, non-targeted fishes are collected along with the target specimens. One bycatch specimen collected between 157 and 167 m represents a new species and the second known species referable to the blenniiform genus *Haptoclinus* Böhlke and Robins, 1974. *Haptoclinus apectolophus* Böhlke and Robins, 1974 was described based on two specimens that were trawled from depths of 174−366 m at Arrowsmith Bank in the northwestern Caribbean. The new species is similar to *Haptoclinus apectolophus* in having the dorsal fin consisting of four parts (three spinous, one soft) and represents a southern range extension for the genus of 9° latitude and an eastern range extension of 17° longitude. In this paper we describe the new species, compare it with *Haptoclinus apectolophus*, and comment on the familial placement of *Haptoclinus*.

## Materials and methods

The specimen was collected by submersible using the fish anesthetic quinaldine pumped from a reservoir through a tube attached to one hydraulic arm and a suction hose (that uses the same pump as the anesthetic-delivery apparatus) attached to the other arm. The latter empties into a vented plexiglass cylinder attached to the outside of the sub. At the surface, the fish was measured, photographed, tissue sampled (right eye removed), and preserved. It was later photographed to document preserved pigment pattern and x-rayed with a digital radiography system. Counts and measurements included in the description are those described for *Haptoclinus apectolophus* by Böhlke and Robins (1974). Measurements were made to the nearest 0.1 mm with an ocular micrometer fitted into a Wild stereomicroscope. Institutional abbreviations follow Sabaj Pérez (2012).

## Results

### Haptoclinus dropi sp. n.

urn:lsid:zoobank.org:act:3091C7AF-C686-4317-AD7E-43D8C8D86357

http://species-id.net/wiki/Haptoclinus_dropi

**Figure 1.**
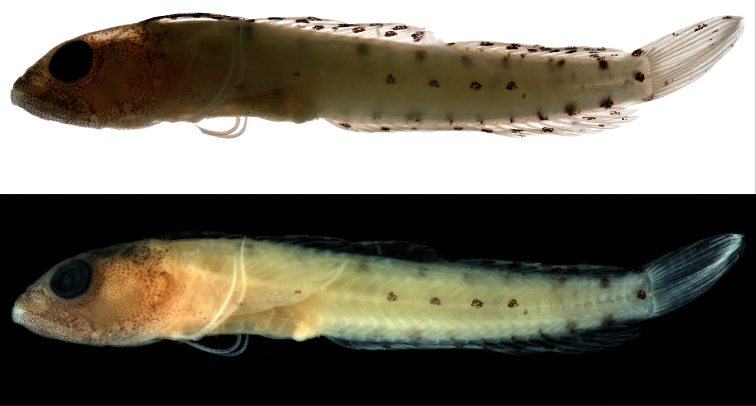
*Haptoclinus dropi*, sp. n., holotype, USNM 414915, 21.5 mm SL, female. Both photographs were taken after the fish was in preservation for several months, the top image against a white background, the bottom against a black background. Photographs by Ian Silver-Gorges.

**Figure 2.**
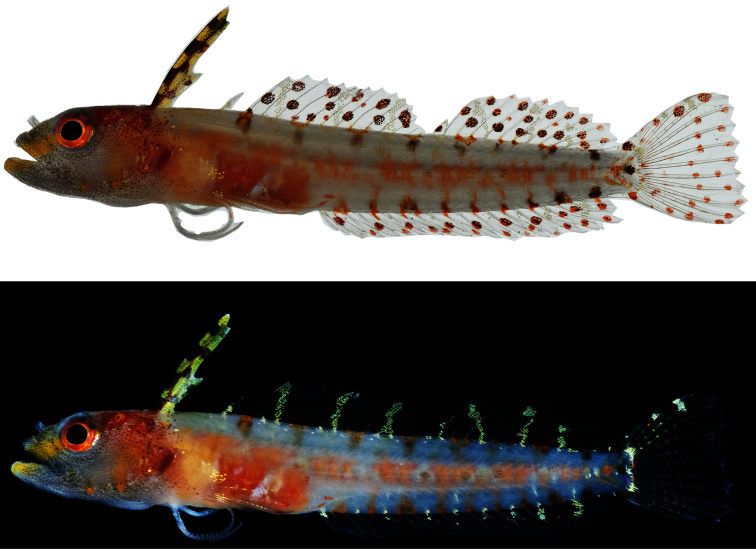
*Haptoclinus dropi*, sp. n., holotype, USNM 414915, 21.5 mm SL, female. Both photographs were taken soon after the fish was captured, the top image against a white background, the bottom against a black background.

Figs 1 –2 Four-fin blenny 

#### Type locality.

Curaçao, southern Caribbean

#### Holotype.

USNM 414915, 21.5 mm SL, female, *Curasub* submersible, sta. 12-7, southern Caribbean, Curaçao, east of downline off Substation Curaçao dock, near 12°05.069'N, 68°53.886'W, 157−167 m, quinaldine, 13 Aug 2012, D. R. Robertson, A. Schrier, B. Brandt, C. Castillo.

#### Diagnosis.

A species of *Haptoclinus* distinguished from its congener by the following combination of characters: dorsal-fin elements III-I-XIII, 12; anal-fin soft rays 19; pectoral-fin rays 12; precaudal vertebrae 12; first dorsal-fin spine longer than second dorsal-fin spine; scales absent; posterior nostril without fleshy flap; and trunk, dorsal- and anal fins with spotted pigment pattern in preservative.

#### Description.

Dorsal-fin elements: III-I-XIII, 12; anal-fin elements II, 19; ultimate pterygiophore of dorsal and anal fins supporting a single segmented soft ray. Pectoral-fin rays 12, 12. Pelvic-fin rays I, 3. Segmented caudal-fin rays 7+6, procurrent caudal-fin rays 6+5. All fin rays unbranched. Vertebrae 12+24=36. Three anal-fin pterygiophores anterior to first haemal spine.

Measurements (in mm): head length 6.6, snout length 1.3, eye diameter 1.0, body depth at fourth dorsal-fin spine 3.5, depth at caudal peduncle 1.0, greatest head width 5.4, body width at anus 2.0, width of bony interorbital 0.4, length of upper jaw 2.6, length of caudal peduncle 2.0, distance from snout to origin of dorsal fin 4.1, distance from snout to upper pectoral-fin base 5.9, distance from snout to insertion of pelvic fin 4.4, distance from snout to origin of anal fin 9.2, length of first dorsal-fin spine 4.1, length of second dorsal-fin spine 3.8, length of third dorsal-fin spine 1.5, length of fourth dorsal-fin spine 0.7, length of longest pectoral-fin ray 4.2, length of pelvic fin 4.1, length of longest caudal-fin ray 3.4.

Body without scales or scale pockets. Small, pointed teeth present in both jaws and on vomer and palatines; teeth uniserial except on anterior portion of premaxilla and dentary, where several teeth form an inner row. Anterior nostril with long tube; posterior nostril a simple opening with minute fleshy rim anteriorly but no fleshy flap; posterior nostril situated closer to eye than to anterior nostril. Mouth terminal and jaws equal. Upper edge of maxilla not sheathed by lacrimal when mouth closed; maxilla reaching vertical through posterior margin of orbit. Gill membranes broadly joined across but free of isthmus. Dorsal margin of upper lip free and continuous across tip of snout. Head lacking cirri. Pores of cephalic lateralis system as drawn for *Haptoclinus apectolophus* (Böhlke and Robins 1974: Fig. 3).

Dorsal fin originating on head about half way between verticals through posterior margins of eye and operculum; fin terminating slightly anterior to vertical through base of ultimate anal-fin ray. Small membrane connecting last dorsal- and anal-fin rays to caudal peduncle. First dorsal-fin spine longest, reaching base of seventh spine when depressed. Fourth dorsal-fin spine short, separated by gaps from anterior three and posterior thirteen spines. Low membrane connecting last dorsal-fin spine to first segmented soft ray. First anal-fin spine shorter than second. Membranes of pectoral fin notched; in dorsal portion of fin (dorsal to longest ray), membranes extending from distal tip of one fin ray to distal tip of adjacent fin ray; in ventral portion of fin, membranes extending from distal tip of one fin ray dorsally to point well proximal of distal tip of adjacent fin ray. Ninth pectoral-fin ray (from top of fin, fourth from bottom) longest (broken on left side of holotype), this ray on right side of body reaching posteriorly to vertical through base of third segmented anal-fin ray. Pelvic fin reaching posteriorly to anus when straightened; innermost (third) pelvic soft ray very small, about half length of small (0.5-mm long), pelvic-fin spine. Caudal fin truncate.

Color Prior to Preservation (Fig. 2).—When photographed against a white background (Fig. 2, top), the following visible on the fresh holotype: ground color of body pale grey; side of belly with rectangular-shaped patch of orange-brown pigment with indistinct whitish diagonal bar across center; spinal column with series of eight internal, irregular orange blotches; dorsalmost region of trunk (beneath dorsal fin) and ventralmost region of trunk (above anal fin) each with row of eight to nine orange/brown spots; head grey, densely speckled with fine black melanophores; nape orange-brown, with several yellow blotches; iris orange, grading to yellowish inner ring; yellow bar extending anteroventrally from anteroventral corner of orbit to anterolateral aspect of upper jaw and anterior tip of lower jaw; operculum pale orange, with two yellow-orange spots on lower edge; anterior dorsal finlet (spines I-III) creamy yellow, with four irregular dark brown horizontal cross-bars; second dorsal finlet (spine IV) translucent; remainder of dorsal fin translucent, with two or three irregular rows of round orange-brown spots on both spinous and soft portions; anal fin translucent, with two rows of round orange-brown spots;caudal fin translucent, with row of round, mostly orange spots along dorsal and ventral fin margins and two vertical rows of such spots across posterior third of fin pectoral and pelvic finswithout obvious pigment. When photographed against a black background (Fig. 2, bottom), the following also visible on the fresh holotype: series of long, yellow/white, roughly vertical bars on dorsal fin—one on second finlet (spine IV), three on main portion of spinous dorsal fin, four on soft dorsal fin; bars extending onto dorsal portion of trunk as small white blotches; a series of tiny white spots beneath dorsal-fin base just ventral to white blotches; row of small white spots on trunk just above anal-fin base between dark spots, several extending onto rear of anal fin as short white bars; thin white bar across caudal-fin base; several white spots on outer portion of upper caudal lobe.

Color in Alcohol (Fig. 1).—Trunk pale, central region with midlateral row of four small, rounded blotches of melanophores; additional small blotch present at center of posterior end of caudal peduncle; eight internal blotches of pigment present on dorsal portion of trunk beneath dorsal fin: first blotch beneath origin of main portion of spinous dorsal fin (spine V); last blotch on caudal peduncle; eight similar internal blotches present on ventral portion of trunk above anal fin, posterior markings darker and including a few external melanophores. Head tan, covered entirely with fine melanophores. First dorsal finlet with four dark blotches on membrane between first and second spines; some of this pigment extending onto membrane between second and third spines; second dorsal finlet (spine IV) unpigmented; remainder of fin with two or three rows of small, rounded spots. Anal fin with rounded spots in single row on most of fin, posterior portion of fin with two rows; spots in distal row smaller than those in proximal row. Caudal fin with small pigment blotch on bases of dorsal procurrent rays and another on bases of ventral procurrent rays; remainder of fin mostly pale except with several small, dark markings on dorsal portion of dorsal lobe. Pectoral fin pale, with a few dark spots on membrane between lowermost third and fourth rays. Pelvic fin pale.

**Figure 3.**
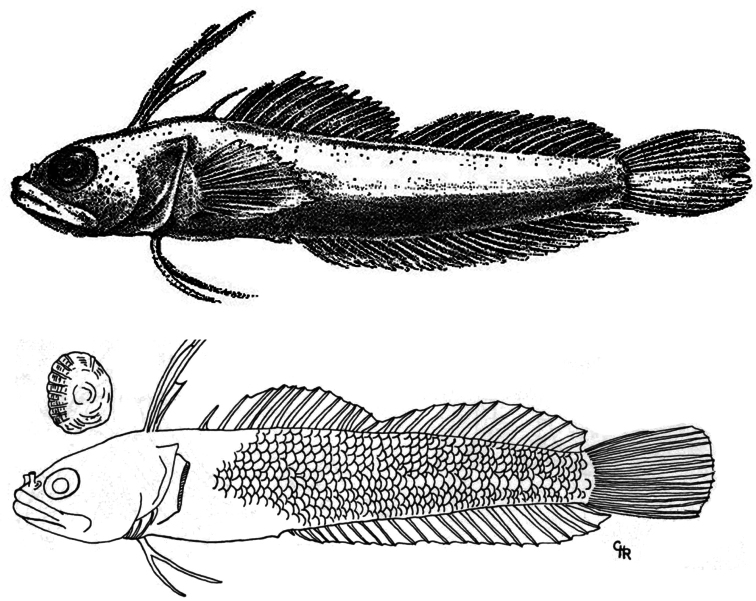
*Haptoclinus apectolophus*, holotype, ANSP 121251, 25.2 mm SL, male. Modified from Böhlke and Robins (1974).

#### Distribution.

Known only from Curaçao, southern Caribbean.

#### Etymology.

The specific name is in reference to the acronym for the Smithsonian Institution’s Deep Reef Observation Project (DROP), which is treated here as a noun in the genitive case. *Haptoclinus dropi* is the first of numerous new species that will be described from DROP submersible research in the southern Caribbean.

#### Common Name.

“Four-fin blenny” is in reference to the configuration of the dorsal fin.

#### Comparisons.

For comparative purposes, counts and measurements of the holotypes of *Haptoclinus dropi* and *Haptoclinus apectolophus* (Fig. 3) are given in Table 1, along with distinguishing features of general morphology. *Haptoclinus dropi* has two fewer dorsal-fin elements than *Haptoclinus apectolophus* (29 vs. 31), the differences occurring in the third spinous dorsal finlet and soft dorsal fin (III-I-XIII, 12 in *Haptoclinus dropi*, III-I-XIII, 14 or III-I-XIV, 13 in *Haptoclinus apectolophus*). *Haptoclinus dropi* also has one or two fewer soft anal-fin rays (19 vs. 20-21), one fewer pectoral-fin ray (12 vs. 13), and one fewer precaudal vertebra (12 vs. 13). The shape of the first dorsal finlet is different in the two species because of differences in relative sizes of the first three dorsal-fin spines: the first dorsal-fin spine is the longest of the three elements in *Haptoclinus dropi* (length of first three dorsal spines 18, 7 and 3% SL, respectively); the second dorsal spine is longest in *Haptoclinus apectolophus* (length of first three dorsal-fin spines 22, 26, and 12% SL, respectively).

*Haptoclinus dropi* and *Haptoclinus apectolophus* have very different preserved pigment patterns. In *Haptoclinus dropi*, the trunk is uniformly pale with a row of external blotches along the lateral midline, a row of mostly internal blotches just beneath the dorsal fin, and a row of mostly internal blotches just above the anal fin. In *Haptoclinus apectolophus*, there is much more pigment on the ventral portion of the body than there is dorsally, and there are no obvious internal or external blotches of pigment. In *Haptoclinus dropi*, the first dorsal finlet has four dark blotches, the fourth dorsal-fin spine is unpigmented, and the remainder of the fin is pale with two or three rows of small dark spots. In *Haptoclinus apectolophus*, the first dorsal finlet is uniformly dark and both the spinous and soft portions of the dorsal fin are peppered with fine melanophores in no apparent pattern. The anal fin is uniformly pale with one or two rows of small dark spots in *Haptoclinus dropi*. In *Haptoclinus apectolophus*, the basal three-quarters of that fin are heavily and uniformly pigmented, and the distal quarter is pale. The caudal fin has dark spots dorsally in *Haptoclinus dropi*, and the pectoral fin has a few dark spots ventrally. In *Haptoclinus apectolophus*, the caudal and pectoral fins lack melanophores. *Haptoclinus dropi* differs from *Haptoclinus apectolophus* in other minor ways: *Haptoclinus dropi* lacks a fleshy flap on the posterior nostril (vs. fleshly flap extending from anterior margin and covering anterior half of nostril) and has a more slender body (body depth 16.3% SL at the fourth dorsal spine vs. 17.9% SL, depth at caudal peduncle 6.6% SL vs. 9.1% SL).

**Table 1.** Counts and measurements of the holotypes of *Haptoclinus dropi*, sp. n.,and *Haptoclinus apectolophus* and distinguishing characters of general morphology. Data for *Haptoclinus apectolophus* are from Böhlke and Robins (1974) or from examination of radiographs of the type (see Comparative Material in the text). Standard length is in mm, other measurements are in % SL.<br/>

	***Haptoclinus dropi***	***Haptoclinus apectolophus***
Catalog number	USNM 414915, Holotype	ANSP 121251, Holotype
SL	21.5	25.2
Dorsal-fin elements	III-I-XIII, 12	III-I-XIV, 13
Total dorsal-fin elements	29	31
Anal-fin elements	II, 19	II, 20
Pectoral-fin rays	12/12	13/13
Pelvic-fin rays	I, 3	I, 3
Segmented caudal-fin rays	7 + 6	7 + 6
Procurrent caudal-fin rays	6 + 5	6 + 5
Vertebrae	12 + 24	13 + 24
Head Length	30.7	29.8
Snout Length	6.0	6.7
Eye Diameter	6.6	7.9
Body depth at 4^th^ dorsal spine	16.3	17.9
Depth at caudal peduncle	6.6	9.1
Greatest head width	25.1	15.9
Body width at anus	9.3	10.3
Bony interorbital width	1.9	2.3
Upper jaw length	12.1	15.1
Caudal-peduncle length	9.3	8.7
Snout to origin of dorsal fin	19.1	21.4
Snout to upper pectoral-fin base	27.4	29.8
Snout to insertion of pelvic fin	20.5	24.2
Snout to origin of anal fin	42.8	46.8
First dorsal-fin spine length	19.1	22.2
Second dorsal-fin spine length	17.7	26.2
Third dorsal-fin spine length	7.0	11.9
Fourth dorsal-fin spine length	3.3	8.3
Longest pectoral-fin ray	19.5	23.0
Pelvic-fin length	19.1	21.4
Longest caudal-fin ray	21.5	20.2
Body squamation	None	Posterior 3/4 scaled
Spotted pigment pattern	Present	Absent
Posterior nostril	No fleshy flap	Fleshy flap anteriorly

## Discussion and conclusions

The configuration of the dorsal fin in *Haptoclinus*, in which the anterior spinous finlet is separated from the main spinous portion by a gap that contains a single isolated spine (the fourth)—thus resulting in a dorsal fin that consists of four parts, is unique among blenniiforms (Böhlke and Robins 1974, Springer 1993). The presence of this configuration in the new species provides solid evidence for its placement in *Haptoclinus*. Furthermore, of the diagnostic characters listed by Böhlke and Robins (1974) for *Haptoclinus* that could be assessed from examination of the preserved holotype and a radiograph of the new species, it deviatesonly in some fin-ray counts and in completely lacking scales. Fin-ray counts often vary intragenerically among species of fishes, but the presence or absence of scales typically does not. However, in the blenniiform genus *Stathmonotus*, five species in the Caribbean and eastern Pacific are naked (Springer 1955), whereas the Caribbean *Stathmonotus stahli* (Evermann and Marsh) is fully scaled (Hastings and Springer 1994). Other characters of the two *Haptoclinus* blennies that exhibit interspecific variability are preserved pigment pattern, number of precaudal vertebrae, configuration of the posterior nostril, and some aspects of morphometry (Table 1). Relative differences in the lengths of the first two dorsal-fin spines in *Haptoclinus dropi* and *Haptoclinus apectolophus* (the first spine is the longest in the former, the second in the latter) result in different finlet shapes in the two species, but whether these reflect interspecific or intersexual differences is unknown. The holotype of *Haptoclinus apectolophus* isa male, and the measurements and illustrations of the first dorsal finlet provided by Böhlke and Robins (1974, also see Fig. 3) are for that specimen. The cleared and stained paratype is disarticulated, and neither sex nor lengths of the first two dorsal-fin spines can be determined. The single specimen of *Haptoclinus dropi* is a female, and no males are currently known.

Familial placement of *Haptoclinus* is uncertain. Böhlke and Robins (1974) assigned *Haptoclinus* to the Clinidae, but they expanded that family to include all blenniiform genera except those in the Blenniidae of Springer (1968). George and Springer (1980) redefined the Clinidae and restricted it to genera previously placed in the Ophiclinidae, Peronedysidae, and Clininae of Hubbs (1952), Penrith (1969) and Springer (1970). All other fishes previously considered clinids in those three publications, plus the more recently described *Haptoclinus*, *Nemaclinus* Böhlke and Springer, *Cottoclinus* McCosker, Stephens and Rosenblatt, and *Xenomedea* Rosenblatt andTaylor, are now placed in the family Labrisomidae [see Hastings and Springer (2009) for a review of the history and current status of blenniiform classification]. In their discussion of *Haptoclinus* affinities, Böhlke and Robins (1974) observed that except for the configuration of its dorsal fin and reduced-but-present squamation, *Haptoclinus* looks like a chaenopsid. The discovery of *Haptoclinus dropi*, which shares with most chaenopsids the complete absence of scales, lends further phenetic support to their observation.

The Chaenopsidae were not defined phylogenetically until Springer (1993) listed synapomorphies of each blenniiform family and Hastings and Springer’s (1994) reviewed the genus *Stathmonotus* and provided a cladistic analysis of chaenopsids. Hastings and Springer (1994) commented on a possible relationship between *Haptoclinus* and the Chaenopsidae and indicated (their Table 1) that of eight chaenopsid synapomorphies, *Haptoclinus* has two: (*2*) *ventral arm of the posttemporal is well separate from the neurocranium* and (*4*) *upper jaw of males extends to or beyond a vertical through the posterior margin of the orbit*. It lacks (*1*) *a long palatine* and (*6*) *a posterior shift in the relative position of the hyomandibula*. They were unable to determine if *Haptoclinus* has (*3*) *no lateral-line ossifications*, (*5*) *a long upper jaw in females*, (*7*) *a sphenotic spine*, and (*8*) *a thin dorsal scapular region that is well separated from the cleithrum*. Citing insufficient information, Hastings and Springer (1994) did not assign *Haptoclinus* to the Chaenopsidae and retained it as an enigmatic member of the Labrisomidae. Hastings and Springer (2009) noted that the Labrisomidae may not be monophyletic and that of the component genera, only *Haptoclinus* and *Nemaclinus* have not been assigned to a labrisomid tribe.

Morphological examination of *Haptoclinus dropi* enables us to add Hastings and Springer’s (1994) characters (*3*) and (*5*) to the list of synapomorphic features shared by *Haptoclinus* and chaenopsids, but characters (*7*) and (*8*) cannot yet be assessed. Hastings and Springer (1994) noted that *Haptoclinus* also shares several derived characters with lineages within the Chaenopsidae, two of which—(*20*) *absence of mandibular pore 1B* and (*21*) *absence of an otic pore*—characterize no other labrisomids. Several characters tabulated by Böhlke and Springer (1975) that separate *Haptoclinus* from other labrisomid genera they examined may warrant additional study because of their presence in some Chaenopsidae. In particular, the complete absence of head cirri is uncommon among blenniiforms but occurs in some chaenopsids (e.g., *Lucayablennius*), and the presence of three (vs. one or two) anal-fin pterygiophores anterior to the first haemal spine occurs in *Lucayablennius* and *Neoclinus* (Springer and Smith-Vaniz 2008). Further comparative morphological work is needed but is hampered by the paucity of *Haptoclinus* specimens. If additional samples of the genus are collected in the future that can be cleared-and-stained and dissected, a more complete assessment of relationships based on morphology can be attempted. The new species has provided the first fresh-tissue sample of *Haptoclinus* for genetic analysis, and future research plans include incorporating genetic data from *Haptoclinus dropi* into a molecular phylogeny of blenniiformes in hopes of shedding light on the phylogenetic affinities of this poorly known genus.

### Comparative material

*Haptoclinus apectolophus*, holotype, ANSP 121251, 25.2 mm SL, male; paratype, ANSP 121252, cleared and stained (disarticulated and in poor condition). Radiograph of holotype examined on the ANSP website: http://clade.ansp.org/ichthyology/FTIP/view.php?mode=details&id=121251.

## Supplementary Material

XML Treatment for Haptoclinus dropi
